# Medical Society of the County of Erie

**Published:** 1897-09

**Authors:** F. C. Gram


### Society Proceedings.

### MEDICAL SOCIETY OF THE COUNTY OF ERIE.

*Special Meeting held July 28, 1897.*

Reported by F. C. GRAM, M. D., Secretary.

THE Medical Society of the County of Erie held a special meeting July 28, 1897, at 8.15 P. M., in the rooms of the Buffalo Academy of Medicine, for the purpose of taking appropriate action on the death of Dr. Edward Storck, who died July 26, 1897, aged 66 years.

An unusually large number of the profession had assembled when the president, Dr. Lucien Howe, called the meeting to order and stated its object. As the secretary had not yet arrived Dr. De Lancey Rochester was selected as secretary pro tempore.

In announcing the death of Dr. Storck the president paid a fitting tribute to his memory, both as a citizen and a physician, especially commending his efforts in elevating the professional standard, as evinced by his work as chairman of the Board of Censors, a position which he filled for many years with credit to himself and the society.

On motion of Dr. JOSEPH HABERSTRO it was decided that the president appoint a committee on resolutions. The following were named: Drs. H. R. Hopkins, Haberstro, W. W. Potter, Hauenstein and Wyckoff.

The committee presented the following memorial:

Forasmuch as it hath pleased Almighty God in His wise providence to take out of this world the soul of our deceased brother, Dr. Edward Storck, we, therefore, unfeignedly and triumphantly declare that for nearly a half century our lamented brother wrought and strove with us in the loyal, honorable and eminent practice of the profession of medicine which he adorned.

That to this society, which he always loved and which gladly honored him with its highest office, he gave for many years, in unstinted measure, of his best thought and most arduous labors, gladly forsaking ease, income and popularity, well knowing that his efforts must bring to himself opposition, misrepresentation and vicious enmity. He wrought manfully for that which made the profession of medicine more clean and of good repute.

That in the wider relation of his chosen profession to the state he carried the same high ideals, the same uncompromising opposition to all that could retard progress or hold back efficiency.

The present medical law of the State of New York, the highest exponent of medical progress, received his hearty sympathy and most efficient support. Therefore,

*Resolved*, That the Medical Society of the County of Erie learns of the decease of Dr. Edward Storck with profound and sincere regret;

*Resolved*, That we tender to his bereaved family our deepest sympathy;

*Resolved*, That we recall his eminent and honorable career with gratitude and inspiration;

*Resolved*, That a copy of this minute be spread upon our records, be offered to the daily press, be suitably engrossed and transmitted to the family of the deceased.

H. R. HOPKINS, JOSEPH HABERSTRO,
JOHN HAUENSTEIN, WILLIAM WARREN POTTER.
C. C. WYCKOFF,

Dated July 28, 1897.

In moving the adoption of the memorial Dr. Hopkins alluded to some of the personal characteristics of the deceased, which showed him in his true manhood, and also referred to his pleasant associations with the departed.

Dr. W. W. Potter, in seconding the memorial, spoke of the work performed by Dr. Storck for his country during the trying period of the civil war, both as a member of the Union Defense Committee and at Fort Porter, and of his many accomplishments beyond the confines of his profession.

Dr. Coakley alluded to Dr. Storck's oratorical abilities, as demonstrated on many appropriate occasions years ago.

Other eulogistic remarks were made by Drs. E. H. Long, Abbott, Thomas Lothrop, Wyckoff, Hauenstein and Conrad Diehl.

A telegram of regret was received by the President from Dr. F. F. Hoyer, of Tonawanda, as follows:

Tonawanda, N. Y., July 28, 1897.

Lucien Howe, M. D.,
*183 Delaware Avenue.*

I am sad at the death of my old friend, Dr. Storck, and regret I cannot be present at the meeting this evening.

FREDERICK F. HOYER.

The resolutions were adopted by a unanimous vote.

It was resolved that the society attend the funeral in a body, meeting at Dr. Dambach's office, at 2.30 p. m., Thursday, June 29, 1897.

The meeting then adjourned.

